# Identification of a 13-Gene Immune Signature in Liver Fibrosis Reveals GABRE as a Novel Candidate Biomarker

**DOI:** 10.3390/ijms26178387

**Published:** 2025-08-28

**Authors:** Wei-Lu Wang, Haoran Lian, Yiling Chen, Zhejun Song, Paul Kwong Hang Tam, Yan Chen

**Affiliations:** 1 School of Pharmacy, Faculty of Medicine, Macau University of Science and Technology, Macau, China; 3220006902@student.must.edu.mo (W.-L.W.);; 2 Precision Regenerative Medicine Research Centre, Medical Sciences Division, Macau University of Science and Technology, Macau, China

**Keywords:** biomarkers, immune landscape, liver fibrosis, GABRE, machine learning, qRT-PCR

## Abstract

Liver fibrosis (LF) poses significant challenges in diagnosis and treatment. This study aimed to identify effective biomarkers for diagnosis and therapy, as well as to gain deeper insights into the immunological features associated with LF. LF-related datasets were retrieved from the Gene Expression Omnibus (GEO) database. Two datasets were merged to generate a metadata cohort for bioinformatics analysis and machine learning, while another dataset was reserved for external validation. Seventy-eight machine learning algorithms were employed to screen signature genes. The diagnostic performance of these genes was evaluated using receiver operating characteristic (ROC) curves, and their expression levels were validated via qRT-PCR experiments. The R language was utilized to delineate the immune landscape. Finally, correlation analysis was conducted to investigate the relationship between the signature genes and immune infiltration. Through the intersection of GEO datasets and Weighted Gene Co-expression Network Analysis (WGCNA), 42 genes were identified. Machine learning methods further narrowed down 13 signature genes (alpha-2-macroglobulin (*A2M*), ankyrin-3 (*ANK3*), complement component 7 (*C7*), cadherin 6 (*CDH6*), cysteine-rich motor neuron protein 1 (*CRIM1*), dihydropyrimidinase-like 3 (*DPYSL3*), *F3*, gamma-aminobutyric acid (GABA) receptor subunit epsilon (*GABRE*), membrane metalloendopeptidase (*MME*), solute carrier family 38 member 1 (*SLC38A1*), tropomyosin alpha-1 chain (*TPM1*), von Willebrand factor (*VWF*), and zinc finger protein 83 (*ZNF83*)), and qRT-PCR confirmed these genes’ expression patterns. Furthermore, these signature genes demonstrated strong correlations with multiple immune cell populations. In conclusion, the 13 genes (*A2M*, *ANK3*, *C7*, *CDH6*, *CRIM1*, *DPYSL3*, *F3*, *GABRE*, *MME*, *SLC38A1*, *TPM1*, *VWF*, and *ZNF83*) represent robust potential biomarkers for the diagnosis and treatment of LF. Among these genes, we first identified *Gabre* as related to LF and expressed in hepatocytes and cholangiocytes. The immune response mediated by these signature biomarkers plays a pivotal role in the pathogenesis and progression of LF through dynamic interactions between the biomarkers and immune-infiltrating cells.

## 1. Introduction

Liver fibrosis (LF), a significant global health challenge, can arise from various factors [1]. If not diagnosed and treated promptly, it can progress to cirrhosis and even liver cancer, posing serious risks to patients’ lives [2]. The global prevalence of liver fibrosis has been steadily increasing over time [3]. Among patients with non-alcoholic steatohepatitis (NASH) cirrhosis, the annual incidence of hepatocellular carcinoma (HCC) is estimated to range from 0.5% to 2.6% [4]. Early and accurate diagnosis and treatment are crucial. However, for LF diagnosis, current limitations include biopsy invasiveness and a lack of accurate non-invasive markers. Well-established diagnostic biomarkers for liver fibrosis, including alpha-2-macroglobulin (A2M) [5] and von Willebrand factor (VWF) [6], have significant drawbacks, such as insufficient specificity and individual variability. Discovering new diagnostic biomarkers can facilitate early-stage diagnosis, allowing for intervention before the disease becomes irreversible. New biomarkers can enhance diagnostic accuracy and reduce the likelihood of misdiagnosis and missed diagnosis. Many new biomarkers can be detected through blood or other body fluid tests, reducing the need for liver biopsy and thus lowering the need for invasiveness procedures. These biomarkers can provide insights into disease progression and treatment response, enabling more individualized treatment plans. Additionally, new biomarkers can offer valuable references for basic research and the development of new therapies for liver fibrosis. Therefore, identifying new efficacious biomarkers for liver fibrosis is imperative.

The gamma-aminobutyric acid type A receptor subunit epsilon (GABRE) is an interesting gene. Current research indicates that GABRE can serve as a diagnostic biomarker for HCC [7]. However, as LF represents a precursor stage to HCC, whether GABRE is associated with LF remains unexplored. Most existing studies on GABRE have predominantly focused on the nervous system [8,9], while its relationship with immune cells during LF requires further investigation.

Research has shown that an imbalanced immune response plays a crucial role in the onset and progression of LF [10]. The liver’s immune system is responsible for maintaining a balance between immunological tolerance and immune response. When this balance is disrupted, it can lead to acute or chronic liver inflammation, presenting as jaundice, liver enlargement, and pain. Thus, gaining a deeper insight into the immunological aspects is vital for improving our understanding of LF pathophysiology.

The progress in artificial intelligence technology and bioinformatics has significantly bolstered advancements in biomedicine. Machine learning, with its robust classification capabilities, has been widely adopted to interpret high-dimensional features from high-throughput data [11]. Additionally, the integration of machine learning with high-throughput microarray analysis has been extensively employed to discover new diagnostic biomarkers. Although numerous studies on diagnostic biomarkers and immune infiltration in LF have been published [10,12], several shortcomings in these studies need to be addressed. First, machine learning offers several advantages over protein–protein interaction (PPI) networks in identifying core targets. It can integrate various data types (e.g., gene expression, clinical, and epigenetic data), providing a more comprehensive analysis [13]. PPI networks primarily depend on protein interaction data, which is relatively limited. Machine learning algorithms can automatically identify and select the most diagnostic or predictive genes through feature selection and dimensionality reduction techniques [13]. This is crucial for handling high-dimensional data, where PPI networks may struggle. Machine learning models can predict new data performance through training and validation [14], identifying key genes and predicting their behavior under different conditions. PPI networks are more descriptive and lack predictive power. Machine learning also offers automation, efficiency, and flexibility [14]. Second, identifying general diagnostic markers for liver fibrosis is advantageous over specific types, offering broad applicability, simplified diagnostic processes, and early detection. Compared to traditional statistical analyses, machine learning approaches offer a more comprehensive analysis for large-scale complex databases, reduce the required costs and professional labor while improving diagnostic accuracy, and facilitate the discovery of novel biomarkers.

**Figure 1:**
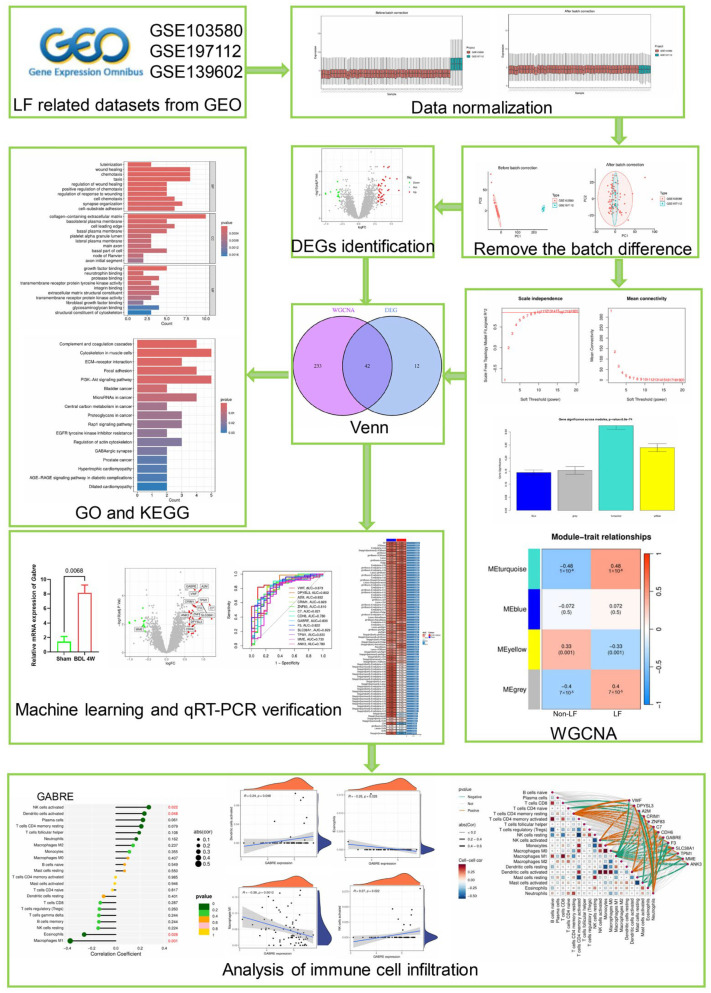
Flow diagram of this research. First, LF-related datasets (GSE103580, GSE197112, and GSE139602) were downloaded from the GEO database, with GSE103580 and GSE197112 serving as the training set and GSE139602 as the testing set. Following standardization and batch effect correction of the GSE103580 and GSE197112 datasets, differential expression analysis using R software identified differentially expressed genes (*n* = 54). Subsequently, WGCNA co-expression analysis was performed to identify disease-associated modules and extract relevant genes (*n* = 275). The intersection of the differentially expressed genes and disease-associated genes yielded a set of overlapping genes (*n* = 42). GO and KEGG enrichment analyses were conducted on these overlapping genes. Then, combining the training set, testing set, and overlapping genes, machine learning was employed. Diagnostic models were constructed using machine learning algorithms, and the optimal model (Random Forest, RF) was selected based on the highest area under the ROC curve. Within the RF model, 13 LF-related signature genes were identified, including GABRE. Subsequently, qRT-PCR experiments were used to validate the expression levels of these genes in liver tissues from bile duct ligation (BDL)-induced cholestatic liver fibrosis. Immune correlation analysis was performed. Additionally, qRT-PCR was used to investigate *Gabre* expression levels in cholangiocyte organoids and hepatocyte organoids.

In our research, we combined metadata from two Gene Expression Omnibus (GEO) databases to conduct differential analyses, develop deep learning models, and examine the immune environment, and the results were validated by a third-party database. We resolved the issue of variability across different data batches. We then investigated methods to uncover the relationship between biomarkers and immune infiltration. Our comprehensive study of the interactions among known biomarkers and their association with the immune environment in liver fibrosis has significantly advanced the field. The detailed analytic workflow is shown in Figure 1.

## 2. Results

### 2.1. Data Preprocessing

**Figure 2:**
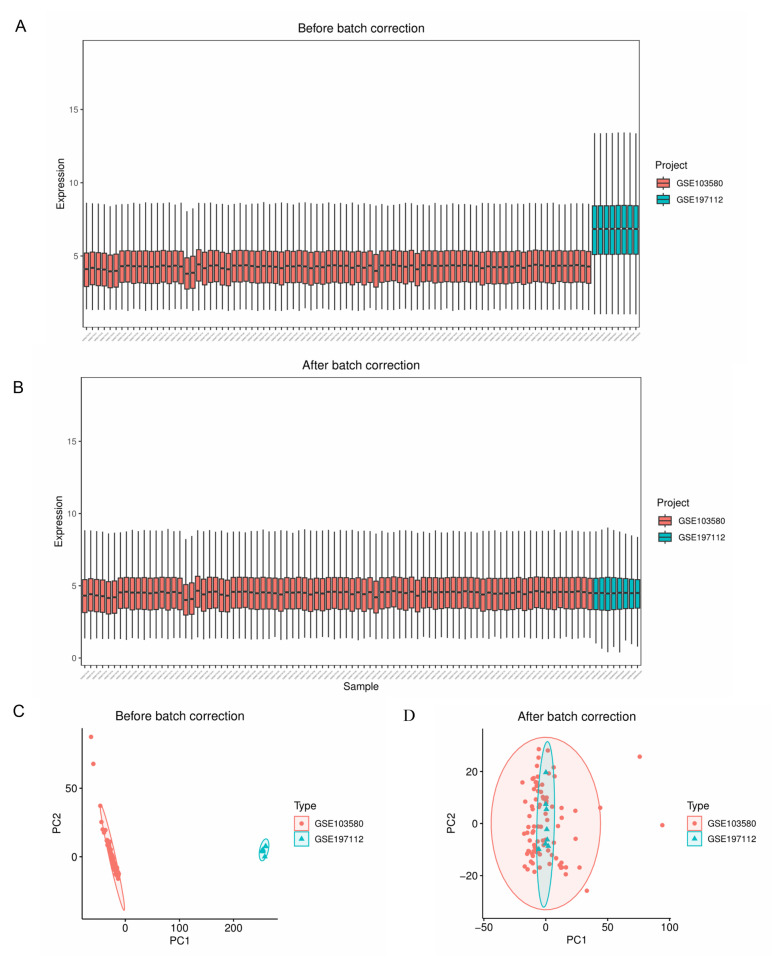
Data normalization and removal of batch effects. (**A**) Box plots before batch correction. (**B**) Box plots after batch correction. (**C**) PCA plot before batch correction. (**D**) PCA plot after batch correction.

The gene expression profiles from the GSE103580 and GSE197112 datasets were acquired after converting gene symbols and normalizing the data (Figure 2A,B). After merging the data and removing inter-batch differences (Figure 2C,D), the metadata file “merge.normalize.txt” was generated from the GSE103580 and GSE197112 cohorts. ComBat was applied for batch effect removal. This metadata included 7396 gene symbols, with 75 LF samples and 19 non-LF samples. The GSE139602 dataset, which contained 29 LF samples and 11 non-LF samples, was used as an independent external validation dataset. Detailed information about these datasets is provided in Table S1.

### 2.2. Hub Gene Screening Using Weighted Gene Co-Expression Network Analysis (WGCNA)

**Figure 3:**
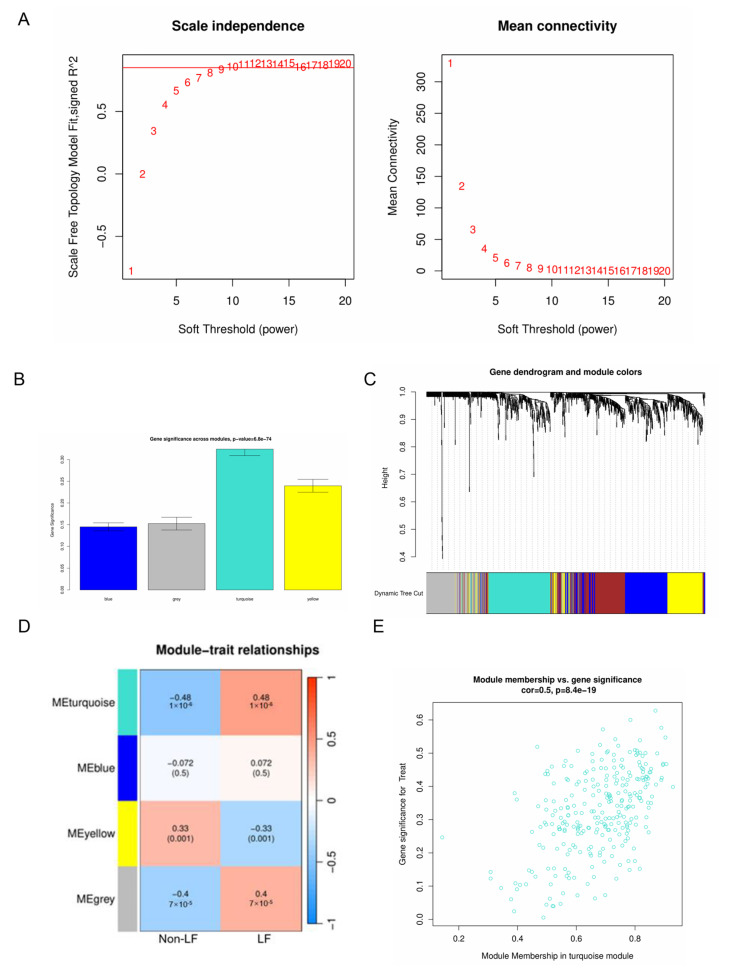
Weighted gene co-expression network analysis (WGCNA). (**A**) Analysis of the scale-free index and the mean connectivity for various soft-threshold powers. (**B**) Gene significance across modules. (**C**) Merged modules under the cluster tree. Different colors represent different modules. (**D**) Module–trait correlations. (**E**) Module membership in the turquoise module vs. gene significance.

After data normalization, the WGCNA method was applied to the GSE103580 and GSE197112 datasets. The selection of an appropriate soft threshold power for WGCNA involved assessing scale-free topology and average connectivity. A power value of 10 was selected based on a correlation coefficient greater than 0.85, as shown in Figure 3A. Utilizing this threshold, a topological overlap matrix was generated. This process led to the discovery of four distinct gene modules, depicted in Figure 3B, with their gene dendrogram and associated module hues, illustrated in Figure 3C. Modules with a strong link to clinical traits typically carry profound biological significance. The turquoise module, in particular, exhibited a notable correlation with liver fibrosis, as indicated in Figure 3D. An in-depth analysis was performed to ascertain the connection between gene significance and the turquoise module. This module’s correlation with gene significance was found to be 0.5, with a *p*-value of 8.4 × 10^−19^, as detailed in Figure 3E. Subsequent investigations focused on the 275 genes within the turquoise module. Comprehensive information regarding the gene symbols, their GS scores, and corresponding *p*-values can be found in the Supplementary Material, under GS and MM.

### 2.3. Identification and Integrative Analysis of DEGs and Intersecting Genes

**Figure 4:**
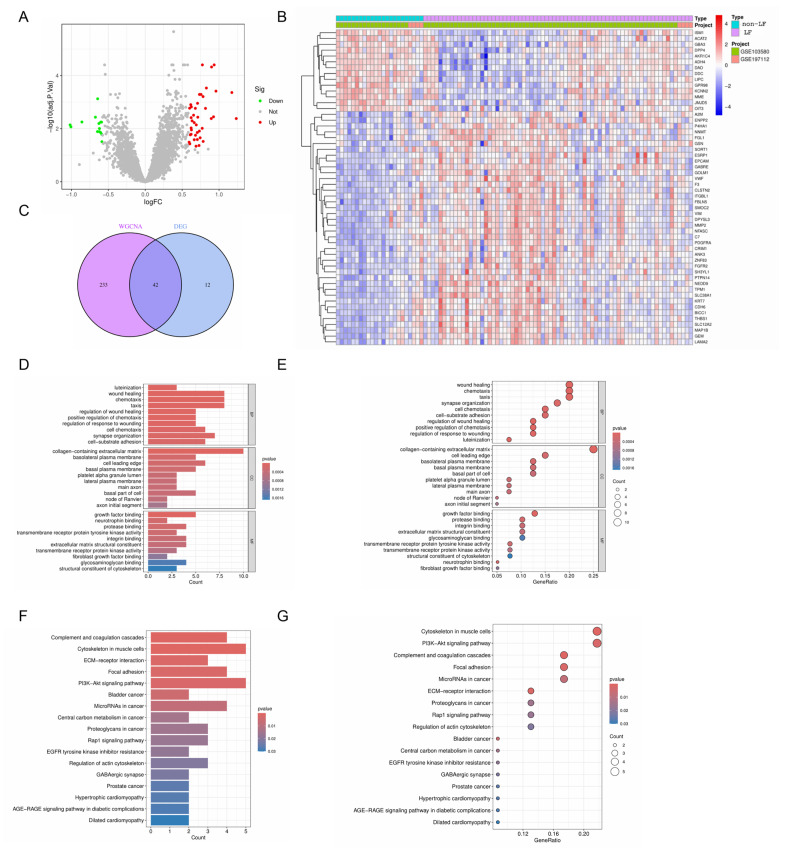
Expression patterns and enrichment analysis of intersecting genes. (**A**,**B**) Volcano plot and heatmap of differentially expressed genes (DEGs). (**C**) Venn diagram of DEGs and WGCNA turquoise module genes. (**D**–**G**) GO and KEGG analysis of intersecting genes.

From the metadata, 54 differentially expressed genes (DEGs) were identified according to the screening criteria of an adjusted *p*-value filter = 0.05 and a logFCfliter = 0.585. Of these DEGs, 40 genes showed significant upregulation, while 14 genes were significantly downregulated (Figure 4A). A heatmap was generated to better illustrate the expression patterns of these DEGs (Figure 4B). Next, we intersected the 54 DEGs with 275 genes from the WGCNA turquoise module using R, yielding 42 intersecting genes (Figure 4C), which are listed in “interGenes.text.” To comprehensively understand the biological processes and pathways associated with these intersecting genes, we performed GO and KEGG enrichment analyses. For the GO analysis, the most significant results are presented using a histogram and bubble diagram (Figure 4D,E). The findings indicated that intersecting genes are mainly involved in biological processes such as wound healing, chemotaxis, taxis, and synapse organization. For the KEGG analysis, the most significant pathways are depicted using a histogram and bubble chart (Figure 4F,G). The KEGG pathways related to LF were primarily associated with the cytoskeleton in muscle cells, the PI3K-Akt signaling pathway, focal adhesion, and the ECM–receptor interaction.

### 2.4. Screening the Signature Genes of LF Using Machine Learning

**Figure 5:**
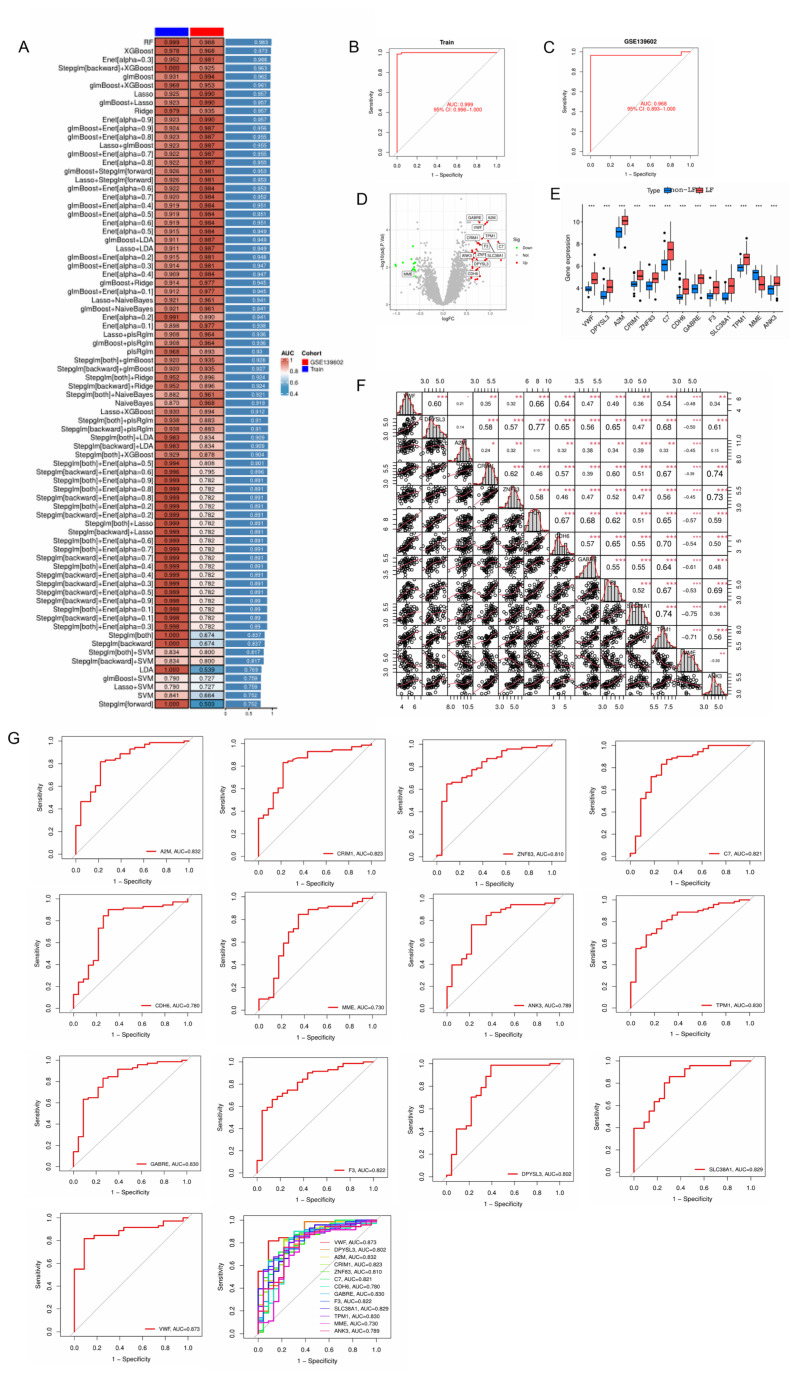
Identification of signature genes by machine learning. (**A**) Performance heatmap of 78 distinct machine learning models. (**B**) ROC curve for the Random Forest (RF) model on the training set. (**C**) ROC curve for the RF model on the independent validation cohort (GSE139602). (**D**,**E**) Expression patterns of the identified signature genes. (**F**) Correlation analysis of signature genes. Statistical significance: * *p* < 0.05, ** *p* < 0.01, *** *p* < 0.001. (**G**) ROC curve for signature genes.

Under the machine learning model, sensitivity and specificity metrics for all 42 genes are presented in ROC.result.xls. To perform machine learning and select the optimal algorithm, as well as identify the genes with the most significant differences between the non-LF and LF groups under the optimal algorithm, we used “interGenes.text,” “merge.normalize.text,” and “GSE139603.normalize.text” as input files. By running R, the “model.AUCheatmap.pdf” file was generated (Figure 5A). From Figure 5A, we observed that the optimal machine learning algorithm was “RF.” The parameters for the RF model are as follows: ntree = 1000; mtry set to default (number of predictors divided by 3 for regression); set.seed (seed = 123); bootstrapping validation with 1000 iterations; node size (nodesize) = 5; variable importance calculation enabled (importance = TRUE). Under the RF algorithm, the AUC (area under the curve) values for the training and test groups were 0.999 (95% CI: 0.996–1.000) and 0.968 (95% CI: 0.893–1.000), respectively (Figure 5B,C). Subsequently, using the optimal machine learning algorithm, we ran R to obtain a list of genes with the most significant differences between the non-LF and LF groups (VWF, DPYSL3, A2M, CRIM1, ZNF83, C7, CDH6, GABRE, F3, *SLC38A1*, TPM1, MME, and ANK3) in “modelGene.list.txt” and the expression levels of these signature genes (Figure 5D,E). The correlations between the signature genes are shown in Figure 5F. The AUC of the ROC curve can evaluate the performance of the machine learning model. The closer the AUC value is to 1, the better the predictive performance of the model. The larger the area under the ROC curve for a gene, the higher the accuracy in distinguishing between control and experimental group samples using that gene. Next, we used “modelGene.list.txt” and “merge.Normalize.txt” as input files to run R and obtain the AUC for each core gene. Since the AUC values for these genes were all greater than 0.7 (Figure 5G), these genes showed high accuracy in predicting LF.

### 2.5. Establishment of an External Liver Fibrosis Model

**Figure 6:**
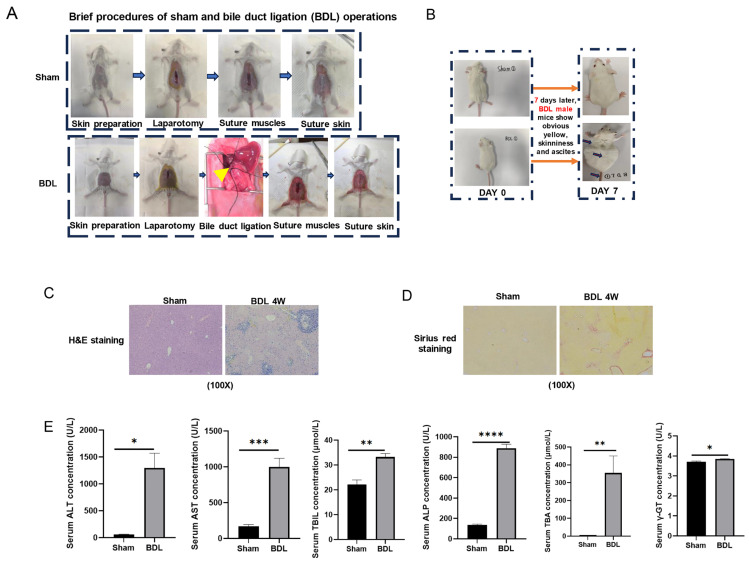
Establishment of a liver fibrosis mouse model. (**A**) Schematic diagram of Sham and bile duct ligation (BDL) surgical procedures. The yellow arrow indicates the ligation site. (**B**) Representative photograph of mice 7 days post-BDL. (**C**,**D**) Histopathological analysis: (**C**) H&E staining and (**D**) Sirius red staining of liver tissues. All the images in (**C**,**D**) were taken using the same magnification, resolution and equipment. The image was captured at an (100×) magnification, with the field of view remaining the same. (**E**) Liver function biomarkers in serum. Statistical significance: * *p* < 0.05, ** *p* < 0.01, *** *p* < 0.001, **** *p* < 0.0001 vs. Sham group.

Because normal Balb/c mice typically develop pronounced and severe liver fibrosis four weeks after bile duct ligation (BDL), we selected liver tissue samples from four-week Sham and four-week post-BDL Balb/c mice as our study subjects. These samples were used for subsequent qRT-PCR experiments to investigate the differences in gene expression between non-LF liver tissue and LF liver tissue. We established an LF model by ligating the common bile duct of mice for four weeks (Figure 6A). Mice with successful model establishment exhibited jaundice and ascites (Figure 6B). H&E staining of the liver showed hepatocyte degeneration and necrosis (Figure 6C), and Sirius Red staining indicated an increase in type I collagen fibers (Figure 6D). Additionally, liver function was abnormal in the liver fibrosis mice, with significantly increased expression of serum liver function-related factors detected using assay kits (*n* = 4) (Figure 6E).

### 2.6. Validation of Signature Genes in an LF Mouse Model and Gabre mRNA Expression in Hepatocyte Organoids and Cholangiocyte Organoids

**Figure 7:**
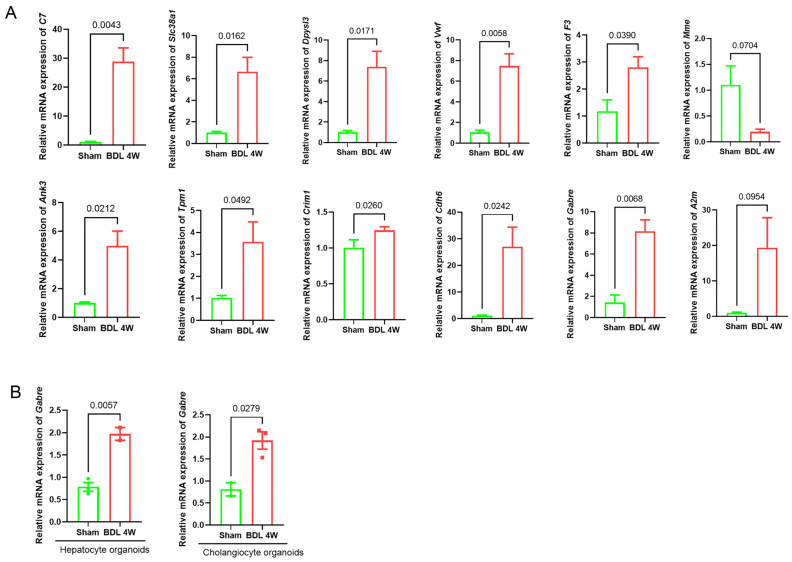
(**A**) Validation of signature gene expression levels in liver tissues of mice with BDL-induced liver fibrosis. Experimental results of qRT-PCR. (*n* = 4). (**B**) *Gabre* mRNA expression in hepatocyte organoids and cholangiocyte organoids. Experimental results of qRT-PCR.

To validate the accuracy of the bioinformatics methods and machine learning algorithms mentioned above, we performed qRT-PCR detection on the signature genes identified by machine learning. The results, which are presented in Figure 7A, were consistent with the predictions made by the machine learning models. To investigate whether *Gabre* is expressed on hepatocytes and cholangiocytes, mRNA from hepatocyte organoids and cholangiocyte organoids were collected. qRT-PCR results showed that *Gabre* was expressed in hepatocytes and cholangiocytes and was upregulated in BDL-induced LF compared to the Sham group (Figure 7B).

### 2.7. Immune Landscape and Correlation

**Figure 8:**
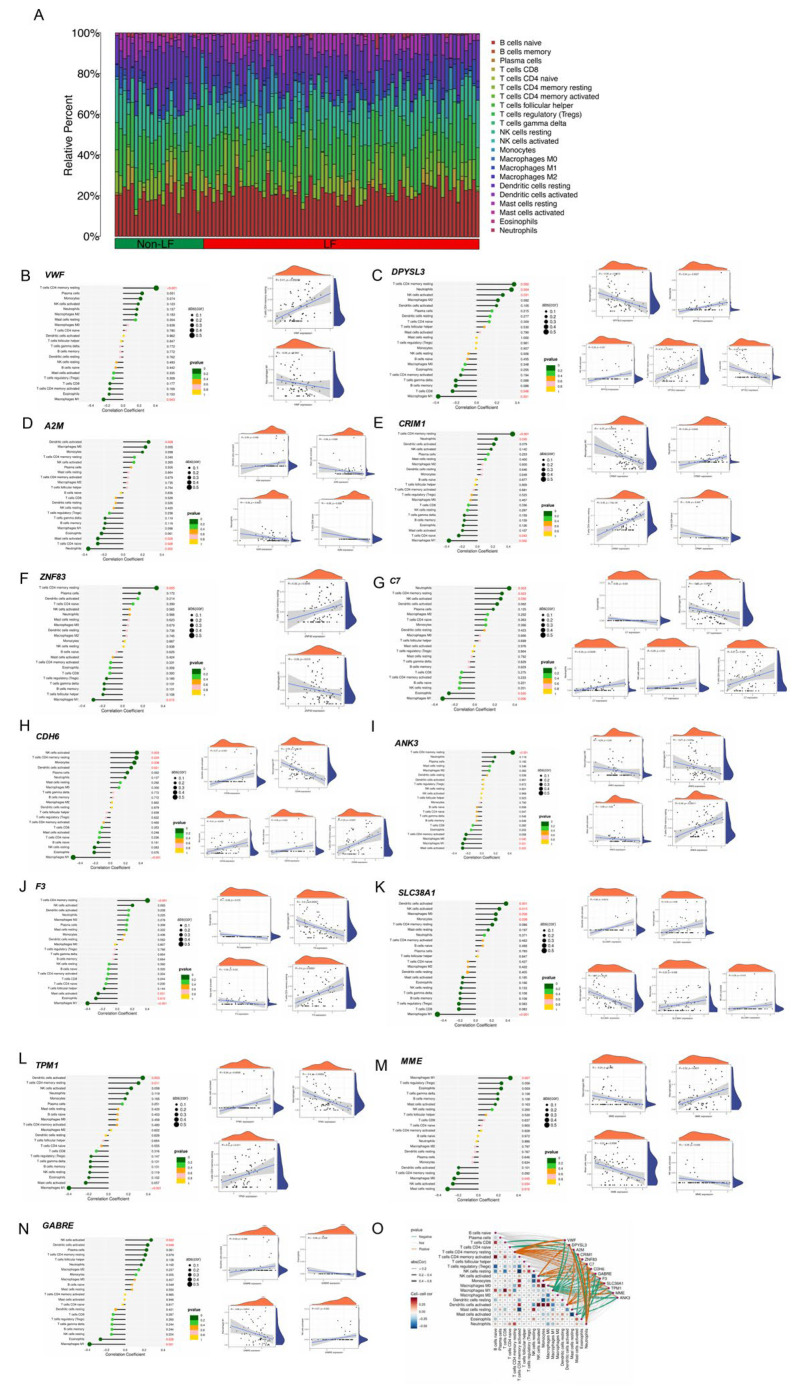
(**A**) Distribution of various cell types between the non-LF and LF groups. (**B**–**O**) The correlation between different types of immune cells and the signature genes.

The immune landscape provided valuable insights into the composition and functionality of immune cells. Initially, we compared the proportion of 22 immune cell infiltrations between the non-LF and LF groups. The results indicated significant differences in the distribution of various cell types between the non-LF and LF groups (Figure 8A). Moreover, the correlation between the signature biomarkers and immune cells was investigated. The signature genes showed little or no correlation with plasma cells, macrophages M2, T cells follicular helper, T cells gamma delta, B cells memory, NK cells resting, B cells naïve, T cells regulatory (Tregs), and T cells CD4 memory activated. However, a significant or moderate correlation was observed between the signature biomarkers and T cells CD4 memory resting macrophages M1 and other immune cells (Figure 8B–N). For the analysis of 13 differentially expressed genes, each corresponding to 22 types of immune cells with multiple *p*-values, we performed false discovery rate (FDR) correction using the Benjamini–Hochberg (BH) method. This procedure was applied to the *p*-values of all 22 immune cell types for each gene, with an FDR control threshold set at 0.05. The comprehensive results are provided in the Supplementary File (FDR.xlsx in Supplementary Materials). These correlations between different types of immune cells and various genes are illustrated in Figure 8O. These findings indicate that different expressions of these signature biomarkers have distinct impacts on the immune infiltration of liver fibrosis.

## 3. Discussion

In this study, 13 signature biomarkers (*A2M*, *ANK3*, *C7*, *CDH6*, *CRIM1*, *DPYSL3*, *F3*, *GABRE*, *MME*, *SLC38A1*, *TPM1*, *VWF*, and *ZNF83*) were identified as significantly correlated with LF through bioinformatics analysis and machine learning. The BDL mouse model and qRT-PCR analysis demonstrated that these biomarkers have excellent discriminatory power in distinguishing LF samples from non-LF samples.

Among the 13 identified biomarkers, VWF, A2M, F3, and MME have been linked to LF. VWF and F3 are essential for blood coagulation, while A2M serves as a key protease inhibitor. Studies have shown that VWF, F3, and A2M are upregulated in LF in both mice and humans [15,16,17,18]. MME, a zinc-dependent metalloprotease, is involved in peptide degradation and cell signaling [19,20]. Research indicates that MME levels are significantly higher in LF patients [20]. MME may contribute to LF progression by modulating ECM degradation and remodeling [19]. Additionally, MME might worsen LF by influencing hepatic stellate cell (HSC) activation and proliferation [19,20]. Our findings align with the existing literature, showing that *VWF*, *A2M*, and *F3* are upregulated in LF in both humans and mice, while MME is downregulated (Figure 5E and Figure 7A). Although *A2m* and *Mme* did not show significant differences in our study due to the small sample size (*n* = 4), their upregulation and downregulation trends are still very evident. Thus, *VWF*, *A2M*, *F3*, and *MME* are crucial biomarkers for liver fibrosis, particularly during active disease phases. Moreover, we found that *VWF* was positively correlated with T cells CD4 memory resting (Figure 8B, FDR.xlsx in Supplementary Materials). *A2M* was negatively correlated with neutrophils (Figure 8D, FDR.xlsx in Supplementary Materials). *F3* was positively correlated with T cells CD4 memory resting and negatively with mast cells activated, macrophages M1, and eosinophils (Figure 8J, FDR.xlsx in Supplementary Materials). *MME* was positively correlated with macrophages M1 and negatively with mast cells resting, NK cells activated, and macrophages M0 (Figure 8M, FDR.xlsx in Supplementary Materials). These findings highlight significant immune cell changes in LF.

DPYSL3 is a cytosolic phosphoprotein expressed in the liver. Currently, DPYSL3 has not been widely reported in the liver-related literature, but limited studies have shown that DPYSL3 expression is upregulated in human HCC cell lines [21]. There is only one article about DPYSL3 in human non-alcoholic fatty liver disease (NAFLD) [22]. Our study directly demonstrates and confirms that *DPYSL3* is upregulated in human and mouse liver fibrosis (Figure 5E and Figure 7A). This is consistent with the study by Hotta et al. [22]. In addition, immunoassays showed that *DPYSL3* was positively correlated with T cell CD4 memory resting state (Figure 8C, FDR.xlsx in Supplementary Materials).

CRIM1 is a glycosylated type I transmembrane protein involved in various biological processes, including tissue development and repair. Our research confirms that CRIM1 is directly related to LF, which is similar to Yang’s report [23]. *CRIM1* expression was upregulated in human liver fibrosis and BDL-induced mouse liver fibrosis (Figure 5E and Figure 7A). Additionally, *CRIM1* was positively correlated with T cells CD4 memory resting and negatively correlated with macrophages M1 (Figure 8E, FDR.xlsx in Supplementary Materials).

ZNF83 is a zinc finger protein primarily involved in transcriptional regulation. Existing data indicate that ZNF83 is mainly expressed in human cells. Our study demonstrates that *ZNF83* is upregulated in human liver fibrosis (Figure 5E). Immunoassays showed that *ZNF83* was positively correlated with T cells CD4 memory resting (*p* < 0.05) and negatively correlated with macrophages M1 (*p* < 0.05) (Figure 8F). However, FDR correction analysis (FDR.xlsx in Supplementary Materials) demonstrated no significant correlations between *ZNF83* and all 22 immune cell types.

C7 is an important component of the complement system, involved in immune responses and inflammation. Previous studies have shown that C7 plays a significant role in liver fibrosis induced by non-alcoholic fatty liver disease [24,25]. Our study indicates that C7 is upregulated in human liver fibrosis samples (Figure 5E), which is consistent with existing research [26]. However, the expression of *C7* in BDL-induced liver fibrosis has not been reported. We are the first to demonstrate that *C7* is upregulated in BDL-induced mouse liver fibrosis (Figure 7A), suggesting that *C7* could serve as a diagnostic marker and potential therapeutic target for cholestatic liver fibrosis. Immunoassays showed that *C7* was positively correlated with neutrophils and negatively correlated with macrophages M1 (Figure 8G, FDR.xlsx in Supplementary Materials).

CDH6 is a calcium-dependent cell adhesion molecule that primarily functions during embryonic development. Currently, there is limited research on CDH6 related to LF [27]. Our study is the first to directly confirm the association between *CDH6* and LF in humans and mice. CDH6 expression was shown to be upregulated during LF (Figure 5E and Figure 7A). Immunoassays first demonstrated that *CDH6* was positively correlated with dendritic cells activated, T cells CD4 memory resting, monocytes, and NK cells activated (Figure 8H, FDR.xlsx in Supplementary Materials). Among these, dendritic cells play a key role in antigen presentation and T cell activation. Studies have shown that the activation of dendritic cells can significantly affect the function of CD4 memory T cells [28].

ANK3 encodes a protein called ankyrin-G, which plays an important role in the stability and function of the cell membrane. Most of the current research on ANK3 has focused on the nervous system [29,30], with few studies on liver fibrosis. Zhang et al. [31] showed that ANK3 expression was upregulated in ccl4-induced liver fibrosis in mice, which is similar to the *Ank3* expression in BDL-induced liver fibrosis in mice in our study (Figure 7A). In addition, our study showed, for the first time, that *ANK3* expression was increased in human liver samples with liver fibrosis compared with non-fibrotic subjects (Figure 5E). Immunoassays showed that *ANK3* was positively correlated with T cells CD4 memory resting and negatively correlated with macrophages M1, macrophages M0, and mast cells activated (Figure 8I, FDR.xlsx in Supplementary Materials).

SLC38A1 (solute carrier family 38 member 1) is a sodium-dependent amino acid transporter primarily involved in amino acid transport and metabolism. Currently, research on SLC38A1 in liver fibrosis is limited, but it is associated with non-alcoholic fatty liver disease fibrosis [32]. In their study, SLC38A1 expression was upregulated in the model group, including both in vivo and in vitro experiments. Our research also indicates that *SLC38A1* expression is upregulated in human liver fibrosis groups (Figure 5E). Interestingly, we also found that *Slc38a1* expression was upregulated in BDL-induced mouse liver fibrosis (Figure 7A), suggesting that *SLC38A1* may be a key diagnostic and therapeutic gene for cholestatic liver fibrosis. Immunoassays first demonstrated that *SLC38A1* was positively correlated with NK cells activated, dendritic cells activated, macrophages M0, and monocytes, and negatively correlated with macrophages M1 (Figure 8K, FDR.xlsx in Supplementary Materials).

TPM1 is a protein involved in cytoskeletal structure and function. Studies have shown that TPM1 expression levels are significantly elevated in hepatocellular carcinoma (HCC) and are associated with tumor invasiveness and patient prognosis [33]. However, there is currently little research directly linking TPM1 to LF. Our study directly confirms that *TPM1* is associated with LF and is upregulated in liver fibrosis samples from both humans and mice (Figure 5E and Figure 7A), providing a stronger theoretical basis for studying *TPM1* and LF. Immunoassays first demonstrated that *TPM1* was positively correlated with dendritic cells activated and T cells CD4 memory resting and negatively correlated with macrophages M1 (Figure 8L, FDR.xlsx in Supplementary Materials).

GABRE (gamma-aminobutyric acid type A receptor subunit epsilon) is a gene associated with gamma-aminobutyric acid (GABA) receptors. Existing research mainly focuses on the role of GABRE in neurological diseases [9,34]. It is mainly involved in inhibitory signaling in the central nervous system. For example, chemical genetic inhibition of GABRE neurons in the preoptic area reduces the heart rate [35]. *GABRE* is not only expressed in the nervous system but also in liver tissue [36]. As an organ of immune tolerance, there is a crossover between immune regulation and GABA signaling in the liver. GABRE may be involved in the following ways: ① Inhibition of intrahepatic immune response: GABA signaling can inhibit the proliferation of T cells and the release of cytokines (such as TNF-α and IFN-γ) and promote the differentiation of regulatory T cells (Treg). GABRE may be involved in maintaining liver immune tolerance and preventing excessive inflammation. ② In the pathological process of liver diseases, such as liver fibrosis, GABRE may be involved in the process by regulating the GABA response of hepatic stellate cells (HSCs). ③ Immune regulation in the gut–liver axis: Metabolites of gut microbiota (such as GABA) enter the liver through the portal vein, which may affect immune homeostasis through intrahepatic *GABRE*^+^ cells and participate in the progression of autoimmune liver diseases (such as primary biliary cholangitis). At present, research on the role of GABRE in HSC activation, immune cell regulation, and LF is limited. Our study is the first to demonstrate and confirm that *GABRE* is upregulated in liver fibrosis samples from both humans and mice (Figure 5E and Figure 7A). Additionally, we are the first to demonstrate a positive correlation between *GABRE* with activated NK cells and activated dendritic cells, as well as a negative correlation between *GABRE* and macrophages M1 and eosinophils (Figure 8N, FDR.xlsx in Supplementary Materials). Significantly, no prior studies have systematically investigated the correlations between these 13 genes and immune cell subsets in the liver microenvironment. Our study provides novel clues and valuable references for future research.

The GSE103580 dataset contains samples from patients with human alcoholic cirrhosis and hepatitis. The GSE197112 dataset contains samples from human fibrotic and non-fibrotic liver tissue. Liver fibrosis is staged into four phases, with stage S4 representing cirrhosis. A normal liver typically progresses from hepatitis to liver fibrosis, then to cirrhosis, and finally to HCC. While LF caused by different etiologies exhibits some similarities in gene expression patterns, there are also significant differences. The development of LF involves multiple biological processes, including inflammatory responses, activation of HSCs, and deposition of extracellular matrix. However, the regulatory mechanisms of these processes may vary depending on the underlying cause. For example, virus-induced hepatitis fibrosis is often accompanied by significant changes in immune-related genes [37,38]; alcohol-related liver disease fibrosis may involve dysregulated expression of genes related to oxidative stress and lipid metabolism [39]; and non-alcoholic fatty liver disease (NAFLD)-associated fibrosis is typically accompanied by disturbances in lipid metabolism and activation of inflammatory signaling pathways [40]. Although some biomarkers can be used for diagnosing liver fibrosis across different etiologies, the applicability of a single biomarker is often limited. Therefore, in clinical practice, combining the detection of multiple biomarkers is frequently employed to improve diagnostic accuracy. Meanwhile, the varying etiologies of LF may potentially impact biomarker discovery and the generalizability of research findings. This is because different etiologies of LF involve distinct pathogenic mechanisms, which can lead to differences in biomarker expression levels and diagnostic performance. Furthermore, certain biomarkers may be effective for specific etiologies but perform poorly in others. A “one-size-fits-all” approach cannot be applied to extend a particular biomarker to all liver disease patients, thereby limiting its clinical utility. To enhance the generalizability and clinical value of research findings, multi-omics data integration should be considered, and biomarker panels should be selected based on the patient’s specific etiology in clinical practice.

The Gene Ontology (GO) findings emphasize the broad and collaborative biological activities engaged in by these central biomarkers. These activities encompass processes such as healing of wounds, cellular chemotaxis, and synaptic assembly (Figure 4D,E). Additionally, the KEGG pathway analysis identified significant pathways linked to biological advancement, including muscle cell cytoskeleton, the PI3K-Akt signal transduction pathway, cell matrix adhesion, and interactions between extracellular matrix receptors (Figure 4F,G). These pathways and processes are crucial in the emergence and progression of LF, positioning them as essential conduits for therapeutic interventions and pharmaceutical research [41].

In the optimal Random Forest (RF) model, we employed bootstrapping (1000 repetitions) as the core internal validation strategy. This approach yielded a notably high mean AUC value (0.999, 95% CI: 0.996–1.000; Figure 5B), an outcome potentially influenced by the limited sample size. To rigorously assess model generalizability, we conducted additional validation using the independent GSE139602 dataset. The consistently high AUC obtained on this external validation set (0.968; Figure 5C) provides unbiased evidence for the model’s robust performance beyond the original dataset. Nevertheless, these elevated AUC values warrant careful interpretation. Potential contributing factors include (1) exceptionally strong model fitting, (2) high similarity in data distributions, (3) the potential presence of undetected overfitting, and (4) high model stability.

It is noteworthy that in this study, the results for the 78 models are relatively independent (the corresponding machine learning code is available on GitHub https://github.com/1270975323/machine-learning--1 (accessed on 16 August 2025)). Crucially, even when using only a handful of models, the resulting AUC values were consistent with those obtained using all 78 models. For the optimal model (RF), we employed the bootstrapping method for validation, which effectively assesses the model’s generalization ability and mitigates overfitting risks. Under this optimal RF model, the accuracy reached 82% in the validation cohort (GSE139602) and 95.7% in the training cohort. This high accuracy (>80%) also indicates reliable performance of the optimal model. No synthetic data was generated to increase sample size, as such data could distort the original data distribution, potentially introducing false positives or negatives, and generally exhibits poor reproducibility. Instead, we prioritized validation using a real-world independent cohort (GSE139602) to more reliably evaluate model generalizability.

We explicitly acknowledge that cross-validation was not utilized in the assessment of the optimal model and that the sample size was limited. Future research will therefore focus on more comprehensive validation using larger-scale datasets. Although this preliminary analysis yielded promising results, subsequent studies will incorporate cross-validation strategies to systematically evaluate model performance across different data partitions and further enhance robustness and generalizability.

Other limitations should also be considered. First, we did not use human LF samples for external experimental validation. Second, during the qRT-PCR validation phase, the sample size was small (*n* = 4), resulting in only noticeable trends rather than statistically significant differences between the control and experimental groups for A2M and MME, and a larger animal cohort would strengthen conclusions. Third, the detailed mechanisms by which these signature biomarkers impact inflammatory and immune responses, leading to the development of liver fibrosis, remain unclear. In addition, regarding *GABRE*, our study lacked in-depth experimental validation (e.g., knockdown/overexpression of *GABRE* in HSCs or other liver models) to establish causality rather than correlation. Although our study suggests that *Gabre* is expressed in hepatocytes and cholangiocytes, it is not known whether *GABRE* is expressed in HSCs or in immune cells within the liver. Perhaps the relationship between *GABRE* and HSCs and immune cells can be used as a follow-up research direction.

## 4. Materials and Methods

### 4.1. Data Collection

For this investigation, the expression profiles of genes were sourced from the Gene Expression Omnibus (GEO) repository, accessible on the National Center for Biotechnology Information’s website (accessed on 10 March 2025), using “liver fibrosis” as the search term. This study included datasets that adhered to the following criteria: (a) derived from human liver examinations; (b) utilized array-based expression profiling; and (c) comprised comparisons between liver fibrosis patients and healthy individuals without liver fibrosis.

### 4.2. Source of Data

Gene expression data, retrieved from the GEO repository, were preprocessed and normalized using R software (version 4.4.1, China, TUNA Team, Tsinghua University). Probes without gene symbols were omitted to maintain data quality. For genes represented by multiple probes, the mean expression level was computed for use as the definitive expression metric. The “limma” package (limma 3.60.6, Melbourne, Australia)’s Normalize Between Arrays function facilitated normalization. Metadata integration involved the cohorts GSE103580 and GSE197112, and the ComBat function from the “sva” package was employed to adjust for batch-to-batch variations, minimizing confounders. By running R, GSE103580 and GSE197112 were merged to produce “merge.normalize.txt”. Box plots depicted normalization states pre- and post-application, while PCA clustering diagrams displayed batch correction impacts.

### 4.3. Identification of Differentially Expressed Genes (DEGs)

Utilizing the “limma” package, the metadata facilitated the pinpointing of genes with differentially expressed genes (DEGs), adhering to the selection benchmarks of an adjusted *p*-value filter of 0.05 and a logFC filter of 0.585. Subsequently, the DEGs were graphically represented through volcano and heatmap plots, constructed using the capabilities of the ggplot2 package (3.5.2, USA).

### 4.4. Construction of the Co-Expression Network and Hub Module Identification Using WGCNA

Weighted Gene Co-Expression Network Analysis (WGCNA) is a comprehensive systems biology technique designed to uncover gene correlation patterns across microarray samples [42]. This method helps identify gene sets with strong covariation, potentially revealing candidate biomarker genes or therapeutic targets by examining the intrinsic connections within the gene set and their association with the phenotype. In this study, WGCNA was applied to the GSE103580 and GSE197112 gene expression matrices using R software (version 4.4.1, Vienna, Austria). The WGCNA co-expression system was established using the WGCNA package (1.73, Los Angeles, CA, USA) [43]. Initially, the batch-corrected expression data file (merge.normalize.txt) was prepared. Subsequently, the “limma” and “WGCNA” packages were installed and loaded. The R script was then executed, ensuring the removal of outlier samples. The optimal power value was determined by combining the fitting index and average connectivity. The correlation matrix was converted into an adjacency matrix, which was then transformed into a topological overlap matrix (TOM). Modules were identified using the dynamic tree cut method. Finally, the relationship between each module and clinical traits was evaluated, with the module showing the highest correlation coefficient selected for further analysis. WGCNA analysis enabled the identification of disease-related modules and genes.

### 4.5. Identification and Enrichment Analysis of Intersecting Genes

We identified the intersecting genes by combining the DEGs with those in the hub modules from WGCNA. Taking “diff.txt” and “module_turquoise.txt” as input files, by running R, we obtained “interGenes.txt”. Next, we utilized the “ClusterProfiler” package (4.12.6, Guangzhou, China) to analyze the biological functions and pathways of these intersecting genes, conducting enrichment analysis for Gene Ontology (GO) and Kyoto Encyclopedia of Genes and Genomes (KEGG).

### 4.6. Screening of Candidate Diagnostic Biomarkers Using Machine Learning

Machine learning algorithms analyze and learn patterns from historical data to build mathematical models, which are then used to predict or classify new data [44]. To perform machine learning, we first ran R with the machine learning input files “merge.normalize.txt,” “GSE139602.normalize.txt,” and “interGenes.txt” in one folder. The files “data.test” and “data.train” were obtained. Then, we took “data.test,” “data.train,” “refer.ML.R,” and “refer.methodLists.txt” as input files. After running the R language, “model.riskMatrix.txt” was obtained for subsequent use. To successfully run the R language, we first set the parameters of the machine learning model in the code and then set the threshold of the number of genes to “min.selected.var = 5.” Next, the variables were screened according to the model combination of the first machine learning method, and the model was constructed according to the model combination of the second machine learning method. If the variables selected by a machine term method were less than the threshold value, the method resulted in null. We prepared a total of 113 machine learning algorithm models in “refer.methodList.txt”; these 113 machine learning algorithm names are listed in “refer.methodList.xls.” After screening, 78 methods were successfully run in the R language. These 78 machine learning algorithms, including Least Absolute Shrinkage and Selection Operator (LASSO), Random Forest (RF), and eXtreme Gradient Boosting (XGBoost), were used to build multivariate logistic regression models and then calculate the classification probability of each sample based on the logistic regression model. The classification score, risk score, and classification of each sample were predicted according to the gene expression. The variables screened by each machine learning method were extracted, and the area under the curve (AUC) value of each model was calculated. The machine learning models were ranked according to the mean AUC to obtain the best model. Before running R, packages such as “ade4,” “caret,” “mboost,” “e1071,” and “BART” needed to be installed. By dynamically selecting and adjusting the algorithm parameters, the combined optimization framework was able to choose the most suitable optimization algorithm at different stages or on different datasets, thereby improving the training speed and accuracy. Combining multiple algorithms also reduced the limitations of a single algorithm, enhancing the model’s generalization ability and robustness [45]. This step was taken to prevent any potential bias towards the majority class and ensure the integrity of the analysis.

### 4.7. Development of the Bile Duct Ligation (BDL) Mouse Model

A total of *n* = 8, six- to eight-week-old male Balb/c mice were housed in an animal room with controlled temperature and humidity, following a 12-h light/dark cycle (ethical approval number: MUST-FDCT-20241114001). The mice were kept in individually ventilated cages and had free access to standard rodent chow and tap water. Before starting the experiment, the mice were acclimatized for one week. These mice were randomly allocated into 2 groups: Sham (*n* = 4) and BDL (*n* = 4), and confirmed to be in good health by a veterinary assessment prior to the study. Each mouse was first labeled with a unique ID (1–8), and random numbers were generated using the RAND function in Microsoft Excel. Mice were then sorted based on the random numbers, with the first four assigned to the experimental group and the remaining four to the control group. The randomization procedure was conducted by a researcher not involved in subsequent experimental procedures to minimize allocation bias. The investigator who generated the random allocation sequence and assigned the animals to the groups did not participate in any subsequent procedures. The personnel responsible for BDL surgery (During Intervention), conducting the behavioral tests (During Outcome Assessment), and performing the data analysis (During Data Analysis) were all blinded to the group assignments. 

BDL and sham surgeries were performed under Avertin (T48402-500G, Sigma-Aldrich, St. Louis, MO, USA)-induced anesthesia, adhering to the methods reported in the literature [46]. In the sham operations, all procedures were identical except for the ligation of the common bile duct. Each mouse was considered an experimental unit, as treatments were applied individually and measurements were recorded per subject. This study is exploratory in nature, aiming to assess the feasibility of the proposed approach. Therefore, the sample size was set to 4, based on practical constraints and ethical considerations. Despite the limited sample size, appropriate statistical methods were employed to ensure the validity of the finding. Exclusion criteria were predefined as follows: (1) death due to anesthesia or surgical complications; (2) failure to meet the model establishment criteria; (3) development of severe unrelated infections; (4) reaching predefined humane endpoints (weight loss >20% of initial body weight, severe lethargy, or inability to access food or water). 

### 4.8. Serological Testing

Four weeks after BDL surgery, blood was collected from the eyeballs of the Balb/c mice and centrifuged at 3000 rpm for 15 min. All surgical procedures were performed under tribromoethanol anesthesia, with blood collection rigorously limited to ≤30 s, followed by immediate hemostasis and prophylactic antibiotic ointment application. For standardized euthanasia, gradual CO_2_ asphyxiation was administered (initial flow rate: 30% chamber volume/min), with death confirmed by pupil dilation and respiratory arrest prior to cervical dislocation as secondary verification. The serum was then collected for liver function tests. The specific steps were carried out on the fully automatic biochemical analyzer (Chemray 800, RWD Life Science Co., Ltd, Shenzhen, China).

### 4.9. Hematoxylin/Eosin (H&E) and Sirius Red Staining

Dissected liver tissues were fixed in a neutral-buffered formalin (BL539A, Biosharp, Tallinn, Estonia), embedded with paraffin, and cut into 5 μm thick sections for subsequent histological examinations [47,48]. Histological examinations of the liver sections were performed by staining with H&E and Sirius Red (Wuhan Servicebio Technology Co., Ltd, Wuhan, China).

### 4.10. Extraction, Culture, and Identification of Hepatocyte Organoids and Cholangiocyte Organoids from BDL Mice

First, the BDL mice were anesthetized and dissected, and liver tissue was isolated, washed, and cut. Next, the liver tissue was digested by enzymes, the enzyme reaction was terminated by filtration, and the cells were collected using magnetic bead sorting and centrifugation. The cells were seeded in Matrigel and cultured in hepatocyte organoid and cholangiocyte organoid growth medium. Organoids were identified via microscopy, immunofluorescence staining, and gene expression analysis. Hepatocyte organoid and cholangiocyte organoid growth were recorded, and experimental data were analyzed to assess organoid function and stability [49,50,51].

### 4.11. RNA Isolation and Quantitative Real-Time Reverse Transcriptase Polymerase Chain Reaction (qRT-PCR)

The livers from four-week post-BDL male Balb/c mice were chosen (*n* = 4). Primers were obtained from PrimerBank using NCBI (National Center for Biotechnology Information (nih.gov), USA) Gene IDs and species-specific parameters. β-actin was used as an internal control due to its stable expression [52]. All primers showed 90–105% efficiency via standard curve analysis (slope −3.3 ± 0.1, R^2^ > 0.99). The liver tissue samples were subjected to homogenization for the extraction of total RNA, utilizing VeZol (R411, Vazyme Biotech Co., Ltd., Nanjing, China) reagents and the QIAGEN RNeasy Micro Kit (74004, Qiagen, Venlo, The Netherlands). Subsequent to standard cDNA synthesis, the ABI StepOne™ system was employed for quantitative PCR with SYBR Green detection. mRNA levels were quantified using the ΔΔCT method [53]. The primer information is listed in Table S2.

### 4.12. Analysis of the Immune Landscape and Gene Correlation

Understanding the immune landscape is essential for analyzing the composition and activity of immune cells, which are crucial for predicting disease progression and therapy effectiveness. Violin plots, created using the “ggpubr” package (0.6.1, Marseille, France), were used to display variations in immune cell infiltration. The “corrplot” package (0.95, Guangzhou, China and Bratislava, Slovak) was utilized to compute the Pearson’s correlation coefficients for each type of immune cell and the correlation between hub genes and immune cells.

### 4.13. Statistical Methods

Data are presented as the mean ± standard error of the mean (SEM). Statistical analyses were conducted using GraphPad Prism 9.4.0 software (GraphPad Software, LLC, San Diego, CA, USA). Comparisons between two groups were made using *t*-tests. A *p*-value of less than 0.05 was deemed statistically significant. All data points were included in the analysis in vivo experiment. 

## 5. Conclusions

We identified 13 candidate biomarkers for LF, namely *VWF*, *DPYSL3*, *A2M*, *CRIM1*, *ZNF83*, *C7*, *CDH6*, *GABRE*, *F3*, *SLC38A1*, *TPM1*, *MME*, and *ANK3*. Among these genes, we first identified *Gabre* as related to LF and expressed in hepatocytes and cholangiocytes. These biomarkers are involved in important biological processes such as wound healing, chemotaxis, directed movement, and synapse organization. Additionally, we found a significant correlation between these signature biomarkers and infiltrating immune cells. These findings suggest that the immune response plays a crucial role in the pathogenesis of LF, attributed to the interaction between signature biomarkers and immune infiltrating cells.

## Data Availability

Data are contained within the article and Supplementary Materials.

## Supplementary Materials

The following supporting information can be downloaded at https://www.mdpi.com/article/10.3390/ijms26178387/s1.

## Abbreviations

A2M	Alpha-2-Macroglobulin
ANK3	Ankyrin 3
AUC	Area Under the Curve
BDL	Bile Duct Ligation
CDH6	Cadherin 6
CRIM1	Cysteine-Rich Transmembrane BMP Regulator 1
C7	Complement Component 7
DEGs	Differentially Expressed Genes
DPYSL3	Dihydropyrimidinase-Like 3
ECM	Extracellular Matrix
F3	Coagulation Factor III (Tissue Factor)
GABA	Gamma-Aminobutyric Acid
GABRE	Gamma-Aminobutyric Acid Type A Receptor Subunit Epsilon
GEO	Gene Expression Omnibus
GO	Gene Ontology
H&E	Hematoxylin/Eosin
HCC	Hepatocellular Carcinoma
HSCs	Hepatic Stellate Cells
IFN-γ	Interferon Gamma
KEGG	Kyoto Encyclopedia of Genes and Genomes
LASSO	Least Absolute Shrinkage and Selection Operator
LF	Liver Fibrosis
MME	Membrane Metalloendopeptidase
NK	Natural Killer
NASH	Non-alcoholic Steatohepatitis
NAFLD	Non-alcoholic Fatty Liver Disease
PPI	Protein–Protein Interaction
PI3K-Akt	Phosphoinositide 3-Kinase-Protein Kinase B
qRT-PCR	Quantitative Real-Time Reverse Transcriptase Polymerase Chain Reaction
RF	Random Forest
ROC	Receiver Operating Characteristic
SLC38A1	Solute Carrier Family 38 Member 1
SEM	Standard Error of the Mean; Tregs: Regulatory T Cells
TNF-α	Tumor Necrosis Factor Alpha
TPM1	Tropomyosin 1
VWF	Von Willebrand Factor
WGCNA	Weighted Gene Co-expression Network Analysis
XGBoost	eXtreme Gradient Boosting
ZNF83	Zinc Finger Protein 83
