# Supplementary material for: Identification of a 13-Gene Immune Signature in Liver Fibrosis Reveals GABRE as a Novel Candidate Biomarker
Source: Int J Mol Sci. 2025 Aug 28;26(17):8387. doi: 10.3390/ijms26178387 (PMC12429109; doi:10.3390/ijms26178387)
Supplement: Supplementary file 1 [file ijms-26-08387-s001.zip › ijms-3792046-supplementary.pdf]

Table S1. Research data.

Dataset	Platform	Database	Specimen	Detail information
GSE103580	GPL13667	GEO	Liver RNA	Used as a training set, 67 samples of liver fibrosis and 19 samples of non-liver fibrosis.
GSE197112	GPL28576	GEO	Liver RNA	Used as a training set, 4 samples of liver fibrosis and 4 samples of non-liver fibrosis.
GSE139602	GPL13667	GEO	Liver RNA	Used as a testing set, 29 samples of liver fibrosis and 11 samples of non-liver fibrosis.

Table S2. Primer information.

Gene Symbol	Forward primer (5->3)	Reverse primer (5->3)
A2M	GGCAGAATTTCCGCTTAGAGG	CACACACGGACACATTCATCT
ANK3	CTGACGTTCACGAGGGAGTTT	GGGCTTGAGACCAGGTTCA
C7	CAACTGCAAGTGGGACTCCTA	CAGCAACTGAACGCCTTCG
CRIM1	ACTCCATCACCGAGTACGAAG	GATGAGGTTTTCATTGCAGGGT
CDH6	CCCTACCCAACTTTCTCAAACC	GAACGGCTCAGCTCATTCC
DPYSL3	CCGTCTTCTAATCAAGGGAGGG	AATCGTCCACTGTGGTCATTC
F3	AACGTGGTTGTAAAAGACTCACT	GCTTGAGCCTTTCCGATAAGTAA
GABRE	CCTTCAGTGGAGGGTTGGAC	ATTCAGGCGGAGTTAGAGGCT
MME	TCCTGACTATCATAGCGGTGAC	GACGTTGCGTTTCAACCAGC
SLC38A1	GACGTTGCGTTTCAACCAGC	CCTCCTACTCTCCCGATCTGA
TPM1	CAGAAGGCAAATGTGCCGAG	TCCAGCATCTGGTGCATACTA
VWF	CTCTTTGGGGACGACTTCATC	TCCCGAGAATGGAGAAGGAAC
β-actin	GGCTGTATTCCCCTCCATCG	CCAGTTGGTAACAATGCCATGT
